# Insights into Geographic and Temporal Variation in Fatty Acid Composition of Croton Nuts Using ATR-FTIR

**DOI:** 10.1155/2018/4739759

**Published:** 2018-09-03

**Authors:** Nathan W. Bower, Murphy G. Brasuel, Eli Fahrenkrug, Matthew D. Cooney

**Affiliations:** ^1^Chemistry and Biochemistry Department, Colorado College, Colorado Springs, CO 80903, USA; ^2^GIS Technical Director, Colorado College, Colorado Springs, CO 80903, USA

## Abstract

*Croton megalocarpus* seedcake oils from 30 different locations in south central Kenya were analyzed for their fatty acid composition using ATR-FTIR to determine the efficacy of a simple procedure for measuring initial geographic and subsequent temporal variation during five months of seed storage. To our knowledge, this is the first report showing variation in how oils in untreated nuts from different locations change during storage, and how these differences are correlated with local environments. These variations are important to forensic authentication efforts and they provide insights into ways to optimize Croton oil composition.

## 1. Background

*Croton megalocarpus* Hutch. (Euphorbiaceae) is a fast-growing tree indigenous to East Africa with a range extending from moist montane forests to dry savannas [1]. It has seen some use in traditional medicine [2], but recent use has focused on its potential as a local source of biodiesel fuel [3]. In order to optimize performance and minimize maintenance of engines that use biodiesel, a uniform and reproducible oil is desirable [4]. Because of the range of ecological habitats where the trees are found, it seemed there might be differences in the oil composition due to different climatic variables and/or to different varieties/ecotypes of* Croton megalocarpus*. This study was undertaken to determine whether there are differences in the nut oil composition from different locations, due to either varietal differences or habitat variation. It was also undertaken to test the efficacy of ATR-FTIR (attenuated total reflectance Fourier transform infrared) spectroscopy as a faster and less costly method than the wet-chemical and instrument-intensive methods typically used for tracking changes in the oil composition during storage.

Geographic differences of olive oil varietals have been observed and used to help with the authentication of these high-value, registered commodities. These determinations usually require time-consuming sample preparation and analysis with instruments such as gas chromatography–mass spectrometry (GC-MS) or liquid chromatography–mass spectrometry (LC-MS) [5]. Recent research has explored faster and simpler preparation methods such as NIR (near infrared) [6] and ATR-FTIR spectroscopies coupled with chemometric methods such as PLS (partial least squares) regression after centering and scaling each spectrum [7]. Because of the large number of variables involved when using an entire spectrum, spectral processing techniques such as jackknife resampling, cross-validation, and principle components are commonly employed [8]. Subsequent chemometric analysis such as pattern recognition using soft independent modeling of class analogy algorithm (SIMCA) with partial least squares regression (PLSR) has been used to monitor oxidation products and to detect adulterants [9].

While these IR methods require less preparation and analysis time than chromatographic procedures, complex spectral processing and chemometric analyses can often camouflage artifacts and make understanding of the data more difficult for an operator inexperienced with either the software or its interpretation. To minimize these issues, we used standard ATR-FTIR spectral analyses from a portable instrument coupled with known oils to calibrate the spectra and the fatty acid methyl ester (FAME) analyses obtained using GC-MS. Finally, we optimized the choice of wavelengths to simplify the data analyses, making this process commensurate with the simplicity of the ATR-FTIR instrumentation.

## 2. Methods

*Croton megalocarpus* nuts from 30 different locations ranging from approximately 0.4901 N and 35.7434 E to -2.91895 N and 37.5058 E were obtained from Eco Fuels Kenya, Ltd. in the fall of 2017. (For a complete listing, see Supplementary Material, Table 1.) Upon receipt, these were dried at 22°C and 15% RH in paper bags over a period of 10–14 days until the average mass loss per day (attributed to water) fell below 0.5%. At that time the first analyses using cold-pressed oils and ATR-FTIR (see below) were conducted and the nuts were transferred to covered polypropylene containers and stored in a 0 to 1°C refrigerator (RH = 0 to 1%) for subsequent analyses conducted over a 4.5 month period.

Cold (22°C) pressing of individual weighed seeds from the Croton nuts (usually holding three seeds) was accomplished after manually removing and weighing the calyx, shell (ovary), and seed coat (testa). An in-house apparatus consisting of a close-fitting 5 mm diameter piston placed inside of an Al cylinder-cup with a small hole drilled in the side allowed access to the expressed oil. The apparatus was placed in a 10 cm bench vice to provide the necessary pressure. A Pasteur pipet was used to transfer a drop of oil to the diamond window of a Bruker ALPHA Platinum ATR-FTIR. To assure a consistent maximum peak absorbance of 0.295 ± 0.001 (1s) at 1743 cm^−1^, a drop thickness 0.5 – 1 mm was maintained for all analyses.

The ATR-IR spectra were collected using the instrument's default conditions (24 spectra averaged together with data collected every 2 cm^−1^), giving a resolution of 4 cm^−1^. The ATR window was cleaned with isopropanol, allowed to dry, and a background collected every 10–15 min. Spectra were baseline subtracted using the instrument's default settings, and both the raw spectra and the baseline-corrected wavenumber versus absorbance files were transferred to Excel for subsequent data processing. (This was done by clicking on the “history” tab and the “AB” tab in the upper left of the spectrum window. The two columns were copied and pasted into Excel.)

Sixteen known vegetable oils (see Supplementary Material, Table 2) containing 8–24% saturated fatty acids (SFA), 10–85% monounsaturated fatty acids (MUFA), and 2–72% polyunsaturated acids (PUFA) were also analyzed by ATR-FTIR to create linear calibrations for these three components. Although PLS regression of all of the spectra may be used, for simplicity, the percentages of these three components were obtained by plotting the frequency of the cis C=C-H stretch near 3009 cm^−1^ versus the log [% PUFA/% MUFA] to obtain the ratio of these two components. The % PUFA was obtained by plotting the ratio of the absorbances measured at 2922 cm^−1^ and 3009 cm^−1^ versus % PUFA. The % SFA was obtained by subtracting the % PUFA and % MUFA from 100.

The fatty acids fractions were also obtained by solvent extraction of unpressed seeds using dichloromethane. This solvent was chosen for its low water solubility, low vapor pressure, and dipole character to replace the more toxic chloroform-methanol solvent traditionally used to extract lipids. Reagent grade (Fisher) CH_2_Cl_2_ is a single, uniform solvent that does not leave residues for the GC-MS and ATR-FTIR analyses. Grinding pistachios in CH_2_Cl_2_ has been shown to give fatty acid profiles nearly identical to those obtained using Soxhlet extraction [12].

The CH_2_Cl_2_ extraction was accomplished by removing the seed coat and grinding individual seeds in 5 mL of CH_2_Cl_2_ with a glass pestle in a porcelain evaporating dish. The extract was filtered through glass microfiber paper (Whatman 934AH) with three washes of 5 mL CH_2_Cl_2_. The CH_2_Cl_2_ was evaporated in a fume hood using a stream of dry air and the crude oil and protein contents were obtained by weighing the products. The oil was analyzed directly using ATR-FTIR or by GC-MS after conversion to the fatty acid methyl esters (FAMEs). If the oil could not be analyzed immediately, it was stored at -10°C.

Conversion of the fatty acids to FAMES for the GC-MS analyses was accomplished following the European Union's Regulation (EU) 2015/1833 for olive oils [13]. This involved dissolving 0.01 – 0.02 g of oil in 2 mL of heptane, followed by addition of 0.2 mL of methanolic KOH (0.11 g of KOH per mL of dry methanol), shaking for 30 sec, and letting the mixture settle for 2–5 min. A milliliter of the supernatant was then transferred to a capped vial containing anhydrous Na_2_SO_4_. This was shaken and allowed to settle for 5 min to remove any residual water and KOH.

The Na_2_SO_4_-dried heptane-FAME mixture was subsequently diluted (0.025 mL/1.5 mL CH_2_Cl_2_) and analyzed using an Agilent GC-MS (7890A GC and 597C MS) with a 30 m x 250 *μ*m x 0.25 *μ*m HP-5MS column using a temperature program (100°C for 1 min, ramped to 270°C at 3°C/min). This program gave near baseline resolution for all detected FAMEs (C14 to C22) except for C18:3 and cis C18:1, which coeluted (see Supplementary Material Table 4 and Supplementary Material Figure 1). These two were quantified using the areas of their 292.3 and 296.3 peaks obtained from selective ion monitoring. The relative amounts of the other FAMEs were determined by integrating the areas of the peaks in the total ion chromatograms (TICs) after calibrating with known oils prepared and analyzed using the same EU 2015/1833 procedure.

Statistical analysis of the data and plots of the spatial and temporal variation were conducted using Microsoft Excel 2016, Minitab ver. 18.1 (Minitab, Inc., State College PA 16801-3210, USA), and ArcGIS ver. 10.4.1 with Spatial Analyst Extension. Interpolation between points used an inverse square of the distance for the weighting of points.

## 3. Results and Discussion

Table 1 gives the average mass of a complete nut after the initial week of drying and corrected to a mass that includes a full calyx. (Many nuts had only a partial or no calyx.) The ovary and any associated calyx are removed and repurposed, while the seeds (testa + nut meat) are pressed to obtain the oil and a seedcake that may be used as a feed supplement, as the nut meat contains 55.7% crude protein (Carlo Erba C/N analyzer).

**Figure 1:**
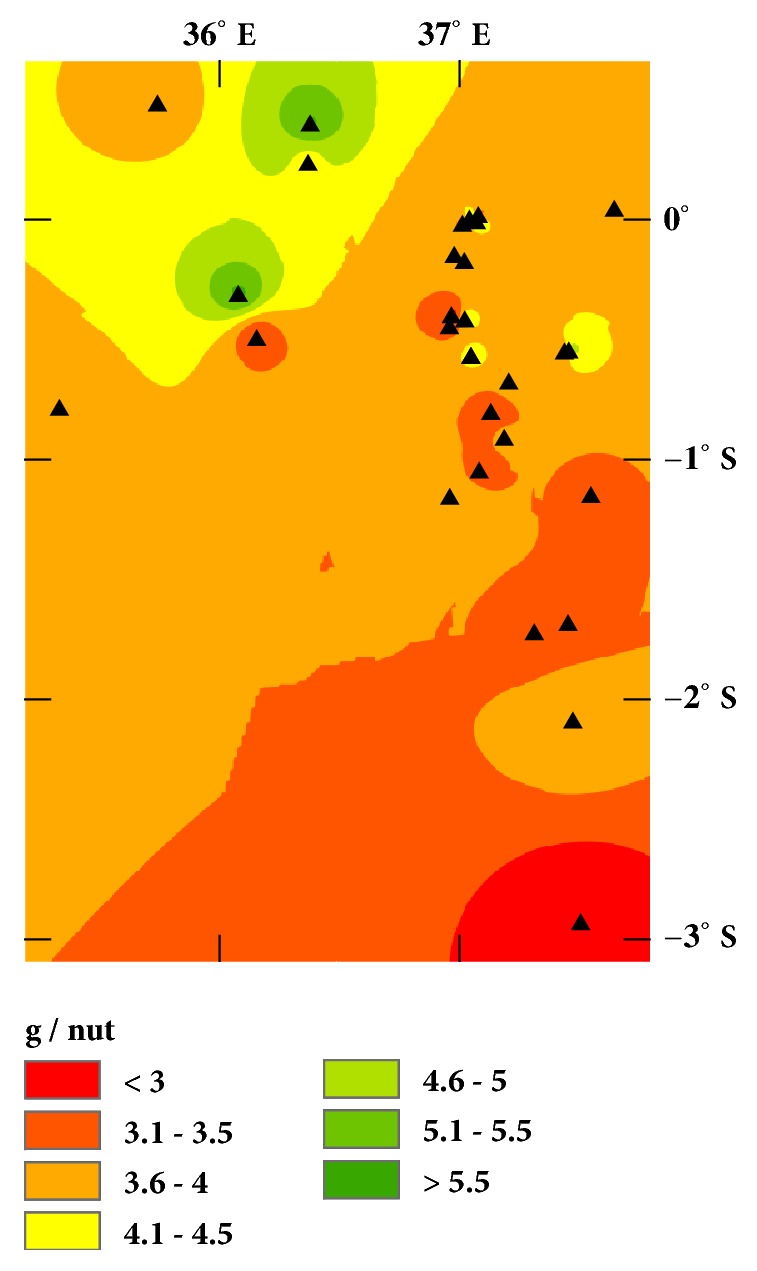
Interpolated map of the average “as received” nut mass at each location.

**Figure 2:**
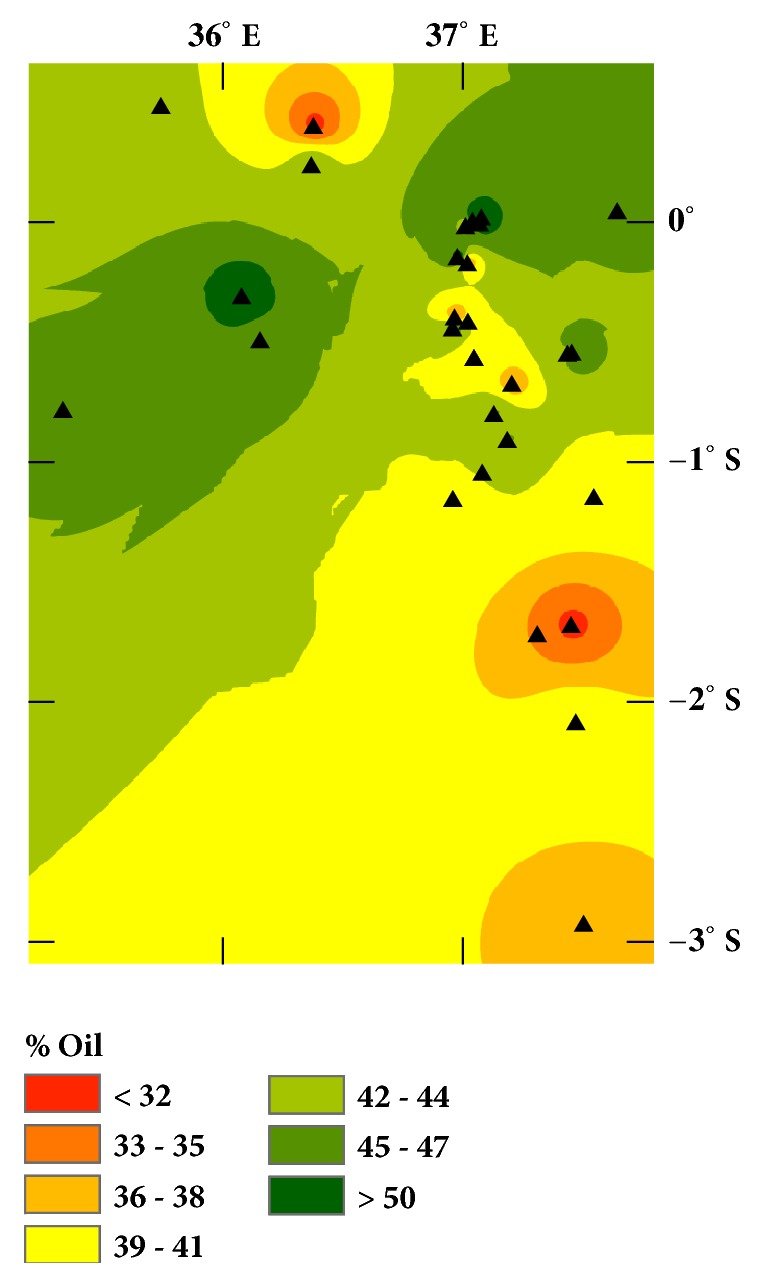
Interpolated map of the mass % oil in the deshelled seed after four months of storage.

Figures 1 and 2 show the variation in the mass and mass percentage of oil extracted from the nutmeat for the various locations. These variables are often associated with different varieties of a plant species, but they are also impacted by the growing conditions, such as temperature, precipitation, and nutrient availability. For example, preliminary analyses of elemental content suggest % Mn and % P (PANalytical XRF) are higher in seeds with higher oil percentages.

**Figure 3:**
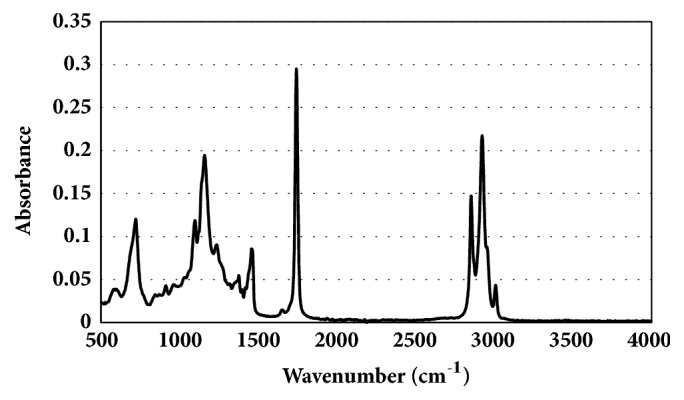
ATR-FTIR spectrum of Croton oil from Sipili, Kenya, before baseline subtraction and normalization to the C=O stretch at 1743 cm^−1^ attributed to the ester carbonyl of the triglycerides.

**Figure 4:**
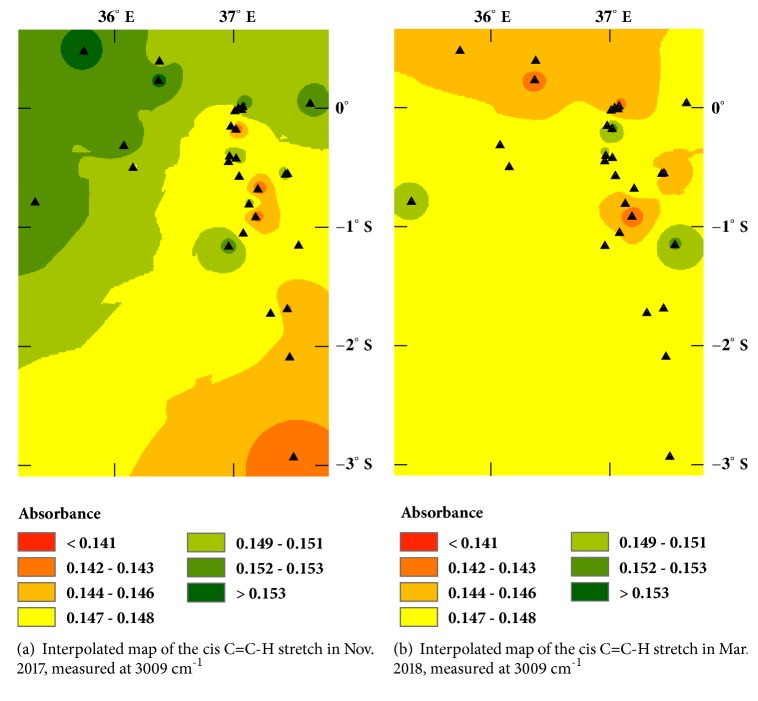


Figure 3 presents a typical spectrum obtained from Croton nut oils. All spectra were similar enough that they appear identical at this scale. However, there are subtle differences that are related to the geography and to the time in storage. Figures 4(a) and 4(b) present the spatial variation observed within the first and last months of the study based on a plot of the absorbance measured at 3009 cm^−1^ after normalizing it to the 1743 cm^−1^ ester peak (set to unity using the baseline-corrected spectra). Sample locations are indicated by black triangles.

Agroclimatic zone maps for the region (see Supplementary Material, Table 5 and Figure 2) indicate the red to orange areas experience mean annual temperatures of 20–22°C, while the green to yellow areas are cooler with mean annual temperatures from 15–20°C [14]. (Some of the interpolated regions without dots contain high montane or arid regions where Croton trees do not grow.) In some instances, the higher absorbances at 3009 cm^−1^ correspond to cool, dry regions which suggests humidity and water availability also play a role in the fatty acid profiles.

**Figure 5:**
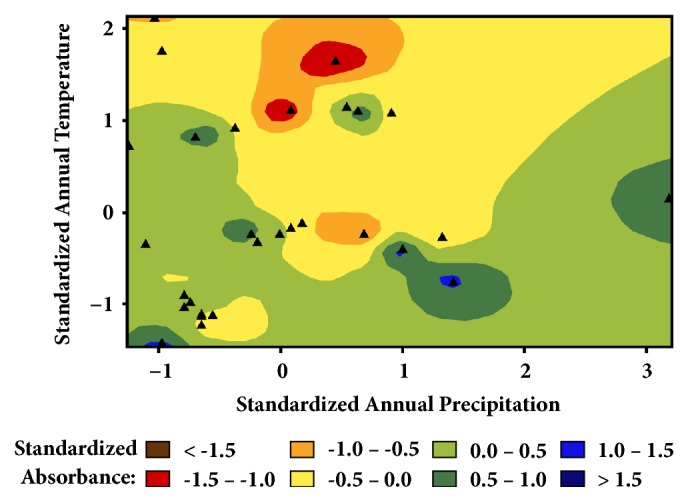
Interpolated map of the March ATR-FTIR standardized absorbance data, which correlates with higher unsaturation, versus the standardized temperature and precipitation.

We expected the geographic variation would reflect varietal/ecotypic differences in the Croton nuts based on similar studies of olive oils [6–8]. However, the correspondence of the variation in Figure 4(a) with the average nut mass (Figure 1), percent oil (Figure 2), and morphology (see Supplementary Material, Figure 3) suggests that the distribution of unsaturation represented by the absorbance in Figure 4(a) may be due to local climate differences. Cooler temperatures and drier environments favor cells producing lower melting oils in order to maintain plasma membrane flexibility [15]. This tendency is illustrated in Figure 5, where the mean annual temperature and precipitation have been standardized (using their z-scores) and are plotted versus the standardized absorbance. Lower temperatures have higher absorbances at 3009 cm^−1^. (Higher absorbances correlate with higher unsaturation;* R*^*2*^ = 0.83,* P* < 0.001,* DF* = 9).

Although less pronounced, average temperatures and average precipitation in the middle of the plot also favor lower levels of saturation. It is the extremes that push the plant cells to make more of the unsaturated fatty acids that give the plasma membranes their flexibility.

The subsequent changes in unsaturation observed during storage at 0°C support this explanation for the variation in Figure 4(a). The average unsaturation increased by a few percent relative to the initial profile, but in a manner that leveled geographic differences. The largest increases in unsaturation (Figure 4(b)) occurred in nuts from sites with higher average annual temperatures (for an interpolated map of temperature and the corresponding changes in the unsaturation, see Supplementary Material, Figure 4). Thus, enzymes that catalyze dehydrogenation reactions at lower temperatures may be involved. If so, then the apparent geographic variation may simply be a plastic response to mean temperature and precipitation differences rather than varietal/ecotype differences [16].

**Figure 6:**
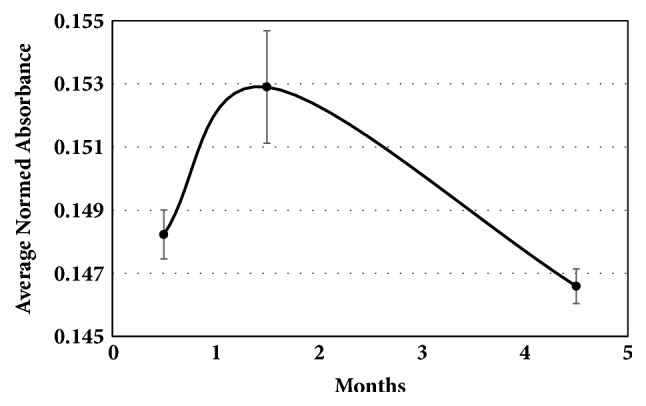
Variation (± 1 SE) in the unsaturation proxy (the normed absorbance at 3009 cm^−1^ due to the cis C=C-H stretch) as measured by ATR-FTIR during the 5 month study.

**Figure 7:**
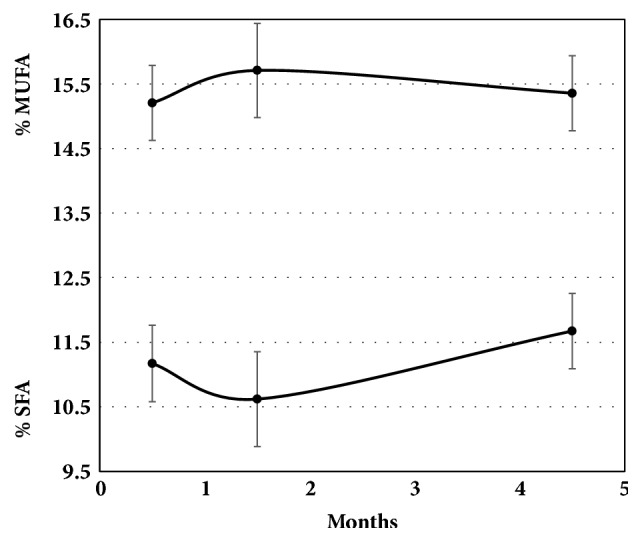
Average mass percentages (± 1 SE) of monounsaturated and saturated fatty acids, calculated from the ATR-FTIR spectra. (Polyunsaturated fatty acids comprise the remainder.)

Based on these observations, we attribute the variation in the different levels of unsaturation (saturated, mono- and polyunsaturated) over time shown in Figure 6 which appeared when Croton nuts were placed in a 1°C refrigerator to the plasticity of living seeds as they respond to a temperature change. This increase only lasted about a month before a slower process that we attribute to chemical oxidation took over that decreased the overall unsaturation. This decrease is similar to what happens with drying of oils like linseed oil [17] or with cold storage of pine nuts [18] and almonds [19]. Based on GC-MS analyses of the extracted oils, over the three-month period from the highest level of unsaturation in Figure 6 to the lowest level at 4.5 months, the saturated fatty acids (primarily C18:0 and C16:0) and the triply unsaturated fatty acid (C18:3) decreased by about 2, 1, and 3%, respectively. At the same time the mono- and di-unsaturated fatty acids (C18:1 and C18:2) increased by about 2.5% each. Figures 6 and 7 summarize the changes in the unsaturation and fatty acid composition as measured by ATR-FTIR in the cold-pressed oils during the five-month study.

Although a frequency shift in the 3009 cm^−1^ peak to lower values (3008 cm^−1^ and below) is expected as the double bonds are broken, only a small shift in the frequency of the peak was observed despite the declining percentage of the polyunsaturated fatty acid, C18:3. This may be because C18:2 was increasing over the same time interval, or the refrigeration slowed the reaction sufficiently that more time is needed to see a noticeable effect. However, extracted oil samples kept in stoppered flasks at room temperature (22°C) for 3 months did exhibit a significant frequency shift to smaller wavenumbers that was largest for those which were the most oxidized (*R*^*2*^ = 0.57,* P* = 0.018,* DF* = 7). Other components that may reflect geographic differences or biological and chemical reactions over time, such as the iodine value and saponification number [20], the peroxide value [21], the free fatty acid (FFA) [22] content, or the trans-fat content [23], may also be measured. However, the components that relate to degradation of the oils were all below 0.1% even at the end of this study. Nuts contaminated by mold did show a jump in the FFA content to around 0.2% of the total oil over the 4.5 months.

Table 2 presents the fatty acid composition from CH_2_Cl_2_-extracted and cold-pressed oils from nuts that were dried and stored long enough to achieve stable values. Differences may be expected between the two methods used for cell disruption and oil extraction (cold-pressing and grinding in CH_2_Cl_2_), as cold-pressed oils are expected to have higher levels of the more fluid components, notably the low molecular weight saturated and the polyunsaturated fatty acids [24, 25]. Thus, solvent extraction data could appear to have come from regions with lower annual temperatures than data obtained using a cold press if the method is not taken into account.

Despite these expectations, there are no significant differences between the fatty acid profiles of the samples that had stabilized in the refrigerator using these two extraction methods. The results from this study are in agreement with literature values for commercial Croton oil from Tanzania (see Table 2), especially given the decrease in C16:0 and increase in C18:2 during cold storage. However, the higher levels of C18:3 in the Kenyan nuts compared to the Tanzanian values are probably real. Although ATR-FTIR is not the best way to obtain accurate oil composition data, the average Croton nut PUFA, MUFA, and SFA values after 4.5 months were 73.5 ± 5% (1s), 15.6 ± 1.3%, and 10.9 ± 5% compared to the GC-MS values of 80.9%, 10.6%, and 8.5% for these composites of fatty acids obtained using a hand press.

## 4. Conclusions

ATR-FTIR provides a faster and simpler method (operators can be easily trained) than GC-MS for monitoring changes in Croton oil composition over time and space. The accuracy and precision of oil component analyses are somewhat lower than what can be provided by a chromatographic analysis. However, the ability to obtain results on-site in a matter of minutes instead of hours, the lack of expensive reagents or toxic waste, and the ability to store spectra that can be revisited and reprocessed at a later time, such as for peroxide or iodine values or with more sophisticated chemometric data analyses [26], are important advantages.

Kenyan* Croton megalocarpus* nut oils were very homogeneous across the geographic range studied despite differences in size and morphology even though these are not plantation trees. Although only a single season was sampled, we think this study is representative of what will occur in other years.

*Croton megalocarpus* oil has high levels of polyunsaturated fats comparable to the levels found in safflower, hemp, and walnut oils. Many of these have been found to offer significant health benefits compared to saturated fats. This study did not find any significant evidence for species or varietal differences that are related to geography, though freshly collected nuts did exhibit subtle differences in unsaturation that could be discerned using ATR-FTIR analyses of cold-pressed oils obtained with a simple hand press.

Because the geographic pattern observed early in the study faded over time during cold storage, the geographic variation may simply indicate an underlying factor such as higher levels of residual water in larger nuts, or it may reflect the plasticity found within this plant species. The portable ATR-FTIR allows analyses to be conducted in the field before these variations have been leveled by the uniformity of storage conditions. These geographic variations are of great interest as scientists try to understand how climate change will affect both natural plant distributions and the yields that may be expected from agricultural crops. In any case, these observations provide a cautionary tale for applications that rely on oil composition obtained via ATR-FTIR to identify the origins and/or adulteration of geographically registered food commodities.

This study also suggests that the relative percentage of unsaturated fatty acids can be increased simply by storing the nuts at a low temperature for a few weeks, potentially increasing the value of the oil for uses beyond the production of biodiesel fuels. ATR-FTIR may also prove useful at a production facility where blending or processing of oils for improvements in engine performance is being conducted.

Finally, we would be remiss if we did not point out that this study was based on only a single season, so results may differ in years with different weather patterns.

## Figures and Tables

**Table 1:** Composition of the nuts.

Component	Mean	1s
Nut (g) _ _^a^	4.16	1.21
Calyx (%)	15.23	2.75
Ovary (%)	48.68	6.83
Testa (%)	14.64	3.57
Nut meat (%)	21.44	5.57
%* of Nut meat:*		
Meal (%)	56.32	14.19
Oil (%)	42.64	5.27
Ash (%)	1.04	0.52

^a^As received, corrected for any missing calyx.

**Table 2:** GC-MS relative percent fatty acid values.

	CH_2_Cl_2_ Extract_ _^a^		Cold Press_ _^a^		Cold Press	Not stated
FA	Mean	± 1s	Mean	± 1s	Wu [10]	Kafuku [11]
C14:0	0.03	0.02	0.02	0.01	0.04	0.10
C16:0	3.49	0.78	3.56	0.73	6.23	6.50
C18:0	4.34	0.84	4.72	0.95	4.37	3.80
C18:1 _ _^b^	9.11	2.01	10.06	2.54	9.95	11.60
C18:2 _ _^b^	76.57	2.61	75.46	3.39	74.31	72.70
C18:3	5.74	1.72	5.39	1.63	3.62	3.50
C20:0	0.17	0.12	0.18	0.11	0.92	
C20:1	0.48	0.29	0.55	0.32		0.90
C20:2	0.07	0.07	0.05	0.04		0.20

^a^Trace levels (< 0.02%) of C16:1, C16:2, C17:0, C17:1, C17:2 and C22:0 were also detected.

^b^Multiple isomers were observed in each chromatogram and these were added together.

## Data Availability

The ATR-FTIR spectral data used to support the findings of this study are included within the appended supplementary material.

## Supplementary Materials

Supplementary Materials The Supplementary Material provides the following additional Tables. (1) Table 1 is a listing of the 30 different sample locations with their coordinates, average nut masses, % oil in the deshelled seed, and length to width ratio. (2) Table 2 is a listing of the known oils used to calibrate the ATR-FTIR analyses. (3) Table 3 is a listing of the baseline-corrected raw and normalized ATR-FTIR absorbance data used to construct Figures 6 and 7. (4) Table 4 contains GC-MS data with a worked example used to construct Table 2. (5) Table 5 contains the GIS interpolated data for the mean annual precipitation and temperature at each sample site and the z-scores of the ATR-FTIR absorbance data. Additional figures are also supplied, including Figure 1, a typical GC-MS TIC; Figure 2, GIS interpolated maps for the temperature and precipitation specific to the sampled tree sites; Figure 3, interpolated map of the nuts' length to width ratios; and Figure 4, interpolated map of the change between December and March in the unsaturation proxy at 3009 cm^−1^.
