# Supplementary material for: Insights into Geographic and Temporal Variation in Fatty Acid Composition of Croton Nuts Using ATR-FTIR
Source: Int J Anal Chem. 2018 Sep 3;2018:4739759. doi: 10.1155/2018/4739759 (PMC6140129; doi:10.1155/2018/4739759)
Supplement: Supplementary Materials — The Supplementary Material provides the following additional Tables. (1) Table 1 is a listing of the 30 different sample locations with their coordinates, average nut masses, % oil in the deshelled seed, and length to width ratio. (2) Table 2 is a listing of the known oils used to calibrate the ATR-FTIR analyses. (3) Table 3 is a listing of the baseline-corrected raw and normalized ATR-FTIR absorbance data used to construct Figures 6 and 7. (4) Table 4 contains GC-MS data with a worked example used to construct Table 2. (5) Table 5 contains the GIS interpolated data for the mean annual precipitation and temperature at each sample site and the z-scores of the ATR-FTIR absorbance data. Additional figures are also supplied, including Figure 1, a typical GC-MS TIC; Figure 2, GIS interpolated maps for the temperature and precipitation specific to the sampled tree sites; Figure 3, interpolated map of the nuts' length to width ratios; and Figure 4, interpolated map of the change between December and March in the unsaturation proxy at 3009 cm−1. [file 4739759.f1.pdf]

Supplementary Material

Table 1 – Site locations, average nut masses, percent oil, percent loss in mass per day, and initial and final normalized absorbances (from Table 5) at 3009.629 cm^{-1}.

				week 1	% Oil in	Average	Stdev
	Location	North	East	g/nut	nut meat	L/Width	L/W
1	Baringo-Kabarnet	0.490107	35.74342	3.6690	41.15	1.23	0.18
2	Bomet	-0.77779	35.33565	3.7170	44.91	1.29	0.22
3	Elementaita	-0.48716	36.15684	3.0710	46.57	1.12	0.08
4	Emali	-2.07969	37.47314	3.9330	39.72	1.16	0.09
5	Equator-Nanyuki	-0.00045	37.0712	4.6390	49.73	1.31	0.21
6	Gachika	-0.40906	37.02309	4.4880	49.98	1.22	0.11
7	Kabiruini-Nyeri	-0.39203	36.96772	2.7970	34.27	1.20	0.08
8	Kihato	-0.14077	36.97915	3.5680	48.89	1.22	0.07
9	Likii	0.024982	37.07917	3.2130	46.87	1.20	0.06
10	Loitoktok	-2.91895	37.50583	2.3680	37.06	1.27	0.13
11	Mahigaini	-0.54286	37.43833	2.9200	42.17	1.21	0.14
12	Makuyu	-0.90258	37.18748	3.5820	44.12	1.22	0.09
13	Maragua	-0.79442	37.13122	3.0160	44.18	1.27	0.18
14	Matanya	-0.01105	37.01289	3.1010	40.46	1.36	0.13
15	Matuu	-1.1407	37.54812	3.2510	38.53	1.19	0.08
16	Mbooni	-1.67413	37.45344	3.4050	31.41	1.37	0.21
17	Meru	0.051472	37.6456	3.7220	45.28	1.04	0.06
18	Muhotetu	0.242523	36.3705	4.3800	43.45	1.16	0.12
19	MUKURWE-INI-A	-0.56094	37.04876	4.1260	42.57	1.23	0.11
20	MUKURWE-INI-B	-0.56094	37.04876	4.2225	37.97	1.35	0.19
21	Mutulani	-1.71186	37.31285	3.2588	36.04	1.24	0.13
22	Mwea	-0.53896	37.45612	5.2130	47.51	1.21	0.12
23	Nakuru	-0.3031	36.08003	5.5670	48.90	1.17	0.10
24	Naro_Moru	-0.16611	37.02125	3.5880	35.73	1.15	0.09
25	Nyeri	-0.43767	36.95969	3.2560	46.61	1.33	0.09
26	Ruiru	-1.14825	36.96045	4.0090	39.97	1.04	0.07
27	Sagana	-0.66814	37.20629	3.8580	35.66	1.32	0.09
28	Sipili	0.406196	36.3796	5.5320	46.20	1.33	0.13
29	Sweet_Waters	0.010703	37.04236	4.7150	50.68	1.00	0.05
30	Thika	-1.03876	37.08338	3.3180	(moldy)	1.22	0.07

Table 2 –Fresh-pressed and/or Commercial Oils Used for ATR-FTIR Calibration

Normed to 100%	1	2	3	4	5	6	7	8	9	10
Fatty Acid	Almond	Brazil	Canola	Cashew	Corn	Filbert	Flaxseed	Hemp	Macadamia	Olive
Caprylic C8:0	0.00	0.00	0.00	0.05	0.00	0.00	0.00	0.00	0.00	0.00
Capric C10:0	0.00	0.00	0.00	0.05	0.00	0.00	0.00	0.00	0.00	0.00
Lauric C12:0	0.00	0.00	0.00	0.05	0.00	0.00	0.00	0.00	0.12	0.00
Myristic C14:0	4.68	0.00	0.00	0.05	0.00	0.00	0.00	0.04	0.98	0.00
Palmitic C16:0	3.32	13.61	7.64	10.13	10.41	5.37	6.58	6.39	8.99	15.65
Palmitoleic C16:1	0.24	0.19	0.00	0.36	0.00	0.21	0.00	0.06	19.33	1.71
Stearic C18:0	38.66	10.05	0.00	8.32	1.82	2.20	4.43	2.37	3.47	2.18
Oleic C18:1	40.36	40.87	63.78	60.78	27.85	78.66	18.51	10.57	65.17	62.98
Linoleic C18:2	12.66	35.24	9.39	20.10	58.52	13.56	17.25	58.47	1.94	15.56
Linolenic C18:3	0.08	0.03	19.20	0.16	1.01	0.00	53.22	13.59	0.00	1.52
Arachidic C20:0	0.00	0.00	0.00	0.00	0.39	0.00	0.00	8.51	0.00	0.41
SFA	41.98	23.66	7.64	18.66	12.62	7.57	11.01	17.31	13.57	18.24
MUFA	40.60	41.06	63.78	61.14	27.85	78.87	18.51	10.63	84.50	64.69
PUFA	25.32	35.28	28.59	20.26	59.53	13.56	70.47	72.06	1.94	17.07
	11	12	13	14	15	16	17	18	19	
	Peanut	Pecan	Pine	Poppy	Safflower	Sesame	Soybean	Sunflower	Walnut	
Caprylic C8:0	0.00	0.00	0.00	0.00	0.00	0.00	0.00	0.00	0.00	
Capric C10:0	0.00	0.00	0.00	0.00	0.00	0.00	0.00	0.00	0.00	
Lauric C12:0	0.00	0.00	0.00	0.00	0.00	0.00	0.00	0.11	0.00	
Myristic C14:0	0.02	3.56	0.00	0.00	0.10	0.00	0.00	0.16	0.00	
Palmitic C16:0	9.41	3.35	5.75	11.00	6.70	9.74	10.36	6.53	9.06	
Palmitoleic C16:1	0.05	1.10	0.04	0.00	0.08	0.11	0.00	0.10	0.00	
Stearic C18:0	2.26	27.40	2.49	2.50	2.40	6.53	4.15	4.38	3.15	
Oleic C18:1	61.60	48.51	32.16	15.50	11.51	41.69	23.83	24.82	18.79	
Linoleic C18:2	26.16	16.07	59.48	68.50	79.06	41.08	52.85	63.27	69.00	
Linolenic C18:3	0.00	0.00	0.09	2.50	0.15	0.21	8.81	0.44	0.00	
Arachidic C20:0	0.50	0.00	0.00	0.00	0.00	0.63	0.00	0.18	0.00	
SFA	12.19	30.75	8.24	13.50	9.21	16.91	14.51	11.37	12.21	
MUFA	61.65	49.26	32.19	15.50	11.59	41.80	23.83	24.92	18.79	
PUFA	26.16	30.64	59.57	71.00	79.21	41.30	61.66	63.71	69.00	
	Coconut oil was dropped as it was too different from the other oils, giving it undue weight in the calibrations.									
	These oils were also used to check the accuracy of the GC-MS FAME preparation and calibration.									

Table 3 – ATR-FTIR baseline-subtracted absorbance data from Nov., Dec., and Mar.

**ATR-FTIR Data from the start of the study**														
	**Nov., 2017 baselined absorbances**													
	**Wavenumbers:**	3019.824	3017.785	3015.746	3013.707	3011.668	3009.629	3007.59	3005.551	3003.512	3001.473	2999.434	2921.95	1743.383
1	Baringo	0.020926	0.025757	0.03128	0.036752	0.041182	0.04357	0.043364	0.040656	0.036104	0.0309	0.026327	0.21623	0.283457
2	Bomet	0.020942	0.02594	0.031564	0.036997	0.041454	0.043992	0.043715	0.040711	0.036083	0.03102	0.026465	0.215193	0.28774
3	Elementaita	0.020188	0.025136	0.03083	0.036408	0.040948	0.043373	0.043021	0.040167	0.035648	0.030609	0.026116	0.215657	0.292222
4	Emali	0.01993	0.024578	0.029945	0.035362	0.039726	0.04205	0.041799	0.038992	0.034488	0.029674	0.025463	0.218312	0.288565
5	Eq-Nanyuki	0.020346	0.025073	0.030501	0.035939	0.04032	0.04267	0.042503	0.039916	0.035584	0.030629	0.026151	0.217387	0.290269
6	Gachika	0.020761	0.025647	0.031174	0.036706	0.041137	0.043468	0.043236	0.040558	0.036169	0.031225	0.026702	0.215812	0.289172
7	Kab-Nyeri	0.020002	0.024825	0.030439	0.036126	0.040758	0.043241	0.042932	0.040006	0.035401	0.030282	0.025701	0.209822	0.288625
8	Kihato	0.020414	0.025362	0.030932	0.036433	0.04087	0.043315	0.043168	0.040472	0.036047	0.03102	0.026419	0.215164	0.291433
9	Likii	0.021909	0.026737	0.032275	0.037853	0.042447	0.044901	0.044602	0.041785	0.037195	0.031956	0.027275	0.213188	0.289492
10	Loitoktok	0.019038	0.023491	0.028487	0.033647	0.037971	0.04023	0.039935	0.037369	0.033313	0.028836	0.024826	0.22401	0.284424
11	Mahigaini	0.021279	0.026227	0.031736	0.037274	0.04181	0.044194	0.043792	0.040848	0.036203	0.030967	0.026252	0.212471	0.29243
12	Makuyu	0.01921	0.023822	0.029173	0.034442	0.038699	0.041174	0.041224	0.038754	0.034594	0.029984	0.02582	0.219854	0.290004
13	Maragua	0.020265	0.025345	0.031041	0.036561	0.041	0.043407	0.043141	0.040318	0.035796	0.030743	0.026195	0.212858	0.290535
14	Matanya	0.019854	0.024647	0.030031	0.035309	0.039557	0.041917	0.041751	0.039073	0.034745	0.029894	0.025428	0.219664	0.291416
15	Matuu	0.020374	0.025106	0.030492	0.035859	0.040256	0.042679	0.042403	0.039594	0.035175	0.030274	0.025858	0.214632	0.290843
16	Mbooni	0.019821	0.024451	0.029672	0.034846	0.039117	0.041483	0.041354	0.038739	0.034435	0.029769	0.025566	0.21849	0.28936
17	Meru	0.021289	0.026257	0.031914	0.037444	0.04189	0.044341	0.044075	0.041201	0.036564	0.031261	0.0265	0.214364	0.291437
18	Muhotetu	0.020818	0.02582	0.031502	0.037079	0.041596	0.044093	0.043839	0.040869	0.036097	0.030872	0.02632	0.212098	0.287208
19	MUKURWE-INI-A	0.02038	0.025151	0.03062	0.036205	0.040752	0.043197	0.042986	0.040235	0.035783	0.030695	0.025961	0.215096	0.291772
20	MUKURWE-INI-B	0.020401	0.024979	0.030086	0.035199	0.039549	0.042056	0.041871	0.039123	0.034838	0.03009	0.025713	0.219389	0.290654
21	Mutalani	0.019968	0.024891	0.030301	0.035672	0.040034	0.042373	0.042138	0.039335	0.034855	0.02998	0.025615	0.213614	0.285802
22	Mwea	0.020304	0.024996	0.030365	0.035706	0.040134	0.042625	0.042486	0.039764	0.035373	0.030569	0.026158	0.216354	0.292021
23	Nakuru	0.021315	0.026366	0.032002	0.037494	0.042078	0.04473	0.044578	0.041659	0.036953	0.031806	0.027181	0.215769	0.292226
24	Naro-Moru	0.019514	0.023948	0.029035	0.034095	0.038237	0.040569	0.040498	0.038102	0.034147	0.029721	0.025685	0.225648	0.289751
25	Nyeri	0.020049	0.024854	0.030276	0.035626	0.039999	0.04251	0.042479	0.039796	0.035326	0.030373	0.02584	0.21695	0.291547
26	Ruiri	0.020953	0.025817	0.031356	0.037032	0.041748	0.044264	0.043869	0.04083	0.036215	0.031195	0.026624	0.211075	0.290793
27	Sagana	0.019564	0.02419	0.029335	0.034346	0.038432	0.040729	0.040666	0.038249	0.034214	0.029789	0.025876	0.223868	0.288037
28	Sipili	0.020263	0.025209	0.030763	0.036263	0.040789	0.043341	0.043186	0.040366	0.035686	0.030465	0.025855	0.214302	0.288138
29	Sweet-Waters	0.021762	0.026839	0.032474	0.03793	0.042303	0.044694	0.044473	0.041605	0.036931	0.031819	0.027265	0.214582	0.288645
30	Thika	0.019476	0.024438	0.030232	0.035969	0.040475	0.042694	0.042199	0.039332	0.034866	0.029891	0.025426	0.213841	0.290327
	**Normalized absorbances:**													
1	Baringo	0.073824	0.090867	0.110352	0.129656	0.145285	0.153709	0.152983	0.143429	0.12737	0.109011	0.092878	0.762832	1
2	Bomet	0.072781	0.090151	0.109696	0.128578	0.144068	0.152888	0.151925	0.141485	0.125401	0.107806	0.091975	0.747873	1
3	Elementaita	0.069084	0.086017	0.105502	0.12459	0.140126	0.148425	0.14722	0.137454	0.121989	0.104746	0.08937	0.73799	1
4	Emali	0.069066	0.085173	0.103772	0.122544	0.137667	0.145721	0.144851	0.135124	0.119516	0.102833	0.08824	0.756544	1
5	Eq-Nanyuki	0.070094	0.086378	0.105078	0.123813	0.138906	0.147002	0.146426	0.137514	0.12259	0.105519	0.090092	0.748916	1
6	Gachika	0.071795	0.088691	0.107804	0.126935	0.142258	0.150319	0.149517	0.140256	0.125078	0.107981	0.09234	0.74631	1
7	Kab-Nyeri	0.069301	0.086011	0.105462	0.125166	0.141214	0.149817	0.148747	0.138609	0.122654	0.104918	0.089046	0.726971	1
8	Kihato	0.070047	0.087025	0.106138	0.125013	0.140238	0.148628	0.148123	0.138872	0.123689	0.10644	0.090652	0.738297	1
9	Likii	0.075681	0.092358	0.111488	0.130757	0.146626	0.155103	0.15407	0.144339	0.128484	0.110386	0.094217	0.736421	1
10	Loitoktok	0.066935	0.082591	0.100157	0.118299	0.133501	0.141444	0.140407	0.131385	0.117124	0.101384	0.087285	0.787592	1
11	Mahigaini	0.072766	0.089686	0.108525	0.127463	0.142974	0.151127	0.149752	0.139685	0.123801	0.105895	0.089772	0.72657	1
12	Makuyu	0.06624	0.082144	0.100595	0.118764	0.133443	0.141977	0.14215	0.133633	0.119288	0.103392	0.089033	0.758107	1
13	Maragua	0.069751	0.087236	0.106841	0.12584	0.141119	0.149404	0.148488	0.138772	0.123207	0.105815	0.090161	0.732642	1
14	Matanya	0.068129	0.084577	0.103052	0.121164	0.135741	0.143839	0.143269	0.13408	0.119228	0.102582	0.087257	0.753782	1
15	Matuu	0.070052	0.086321	0.10484	0.123293	0.138411	0.146742	0.145793	0.136135	0.120942	0.104091	0.088907	0.737965	1
16	Mbooni	0.068499	0.0845	0.102544	0.120424	0.135185	0.143361	0.142915	0.133878	0.119004	0.102879	0.088354	0.75508	1
17	Meru	0.073048	0.090095	0.109506	0.128481	0.143736	0.152146	0.151233	0.141372	0.125461	0.107265	0.090929	0.735541	1
18	Muhotetu	0.072484	0.0899	0.109684	0.129102	0.144829	0.153523	0.152639	0.142298	0.125682	0.10749	0.091641	0.738482	1
19	MUKURWE-INI-A	0.069849	0.086201	0.104945	0.124087	0.139671	0.148051	0.147327	0.137899	0.12264	0.105202	0.088977	0.737206	1
20	MUKURWE-INI-B	0.07019	0.085941	0.103511	0.121103	0.136069	0.144694	0.144058	0.134603	0.119861	0.103525	0.088466	0.754812	1
21	Mutalani	0.069867	0.087092	0.106021	0.124814	0.140076	0.14826	0.147438	0.13763	0.121955	0.104898	0.089625	0.74742	1
22	Mwea	0.069529	0.085597	0.103982	0.122272	0.137435	0.145966	0.14549	0.136168	0.121132	0.104681	0.089576	0.740885	1
23	Nakuru	0.07294	0.090225	0.109511	0.128305	0.143991	0.153066	0.152546	0.142557	0.126453	0.10884	0.093014	0.738363	1
24	Naro-Moru	0.067347	0.08265	0.100207	0.11767	0.131965	0.140013	0.139768	0.131499	0.117849	0.102574	0.088645	0.778765	1
25	Nyeri	0.068768	0.085249	0.103846	0.122196	0.137196	0.145808	0.145702	0.136499	0.121167	0.104179	0.088631	0.744134	1
26	Ruiri	0.072055	0.088781	0.107829	0.127348	0.143566	0.152218	0.15086	0.140409	0.124539	0.107276	0.091557	0.72586	1
27	Sagana	0.067922	0.083982	0.101845	0.119242	0.133427	0.141402	0.141183	0.132792	0.118783	0.103421	0.089836	0.77722	1
28	Sipili	0.070324	0.087489	0.106765	0.125853	0.141561	0.150418	0.14988	0.140093	0.12385	0.105731	0.089731	0.743748	1
29	Sweet-Waters	0.075394	0.092983	0.112505	0.131407	0.146557	0.154841	0.154075	0.144139	0.127946	0.110236	0.094459	0.743411	1
30	Thika	0.067083	0.084174	0.104131	0.123891	0.139412	0.147055	0.14535	0.135475	0.120092	0.102956	0.087577	0.736552	1

ATR-FTIR Data from the middle of the study

	**Dec., 2017 baselined absorbances**													
	**Wavenumbers:**	3019.824	3017.785	3015.746	3013.707	3011.668	3009.629	3007.59	3005.551	3003.512	3001.473	2999.434	2921.95	1743.383
1	Baringo	0.021037	0.025551	0.03071	0.035985	0.040401	0.04291	0.042796	0.040118	0.035847	0.031211	0.02696	0.218942	0.276488
2	Bomet	0.021037	0.025551	0.03071	0.035985	0.040401	0.04291	0.042796	0.040118	0.035847	0.031211	0.02696	0.218942	0.276488
3	Elementaita	0.021618	0.026324	0.031893	0.037491	0.041992	0.044448	0.044225	0.041415	0.036946	0.032007	0.027511	0.215378	0.281613
4	Emali	0.021618	0.026324	0.031893	0.037491	0.041992	0.044448	0.044225	0.041415	0.036946	0.032007	0.027511	0.215378	0.281613
5	Gachika	0.02018	0.024934	0.03041	0.035952	0.040464	0.042961	0.042853	0.04016	0.035762	0.030886	0.026479	0.214826	0.285925
6	Kihato	0.019875	0.02453	0.030016	0.035383	0.039605	0.041924	0.041833	0.039335	0.035066	0.030126	0.025689	0.212559	0.27079
7	Likii	0.019943	0.024689	0.03006	0.035342	0.039612	0.041935	0.041804	0.039388	0.035424	0.030877	0.026634	0.221976	0.291171
8	Loitoktok	0.01794	0.021693	0.025921	0.030054	0.033505	0.03562	0.035688	0.03362	0.030209	0.026503	0.023198	0.186229	0.214926
9	Mahigaini	0.020612	0.025382	0.030812	0.036163	0.040571	0.043074	0.042881	0.040111	0.035718	0.030836	0.026343	0.217089	0.291151
10	Maragua	0.019978	0.024674	0.030032	0.035397	0.039723	0.042059	0.041827	0.039109	0.034771	0.029991	0.025705	0.220803	0.288584
11	Mbooni	0.020894	0.025951	0.03156	0.037031	0.041448	0.043738	0.043373	0.040666	0.03638	0.031408	0.026709	0.215048	0.288679
12	Meru	0.021287	0.026063	0.031705	0.037302	0.041776	0.044165	0.043887	0.041058	0.036469	0.031426	0.026953	0.216295	0.290811
13	Muhotetu	0.020019	0.024864	0.030371	0.035824	0.040254	0.042774	0.042655	0.039867	0.035389	0.030427	0.025879	0.213506	0.291064
14	MUKURWE-INI-B	0.020003	0.024776	0.030308	0.0358	0.040119	0.042348	0.042082	0.039461	0.035227	0.03044	0.026077	0.220362	0.291666
15	Mwea	0.016308	0.020041	0.023968	0.027831	0.031261	0.033362	0.033325	0.031289	0.028126	0.024607	0.021347	0.164762	0.189519
16	Ruiri	0.020319	0.025108	0.030548	0.036068	0.040572	0.042966	0.04277	0.040125	0.03584	0.030992	0.026417	0.217482	0.29274
17	Sipili	0.020003	0.02473	0.030246	0.03585	0.040511	0.043039	0.042696	0.039753	0.035171	0.030198	0.025754	0.216064	0.290497
18	Sweet-Waters	0.020106	0.025186	0.030931	0.036433	0.040674	0.04293	0.042642	0.039849	0.035491	0.030708	0.026289	0.212335	0.281656
19	Thika	0.020843	0.025699	0.031353	0.03692	0.041379	0.04389	0.043768	0.04099	0.036502	0.031554	0.027124	0.214386	0.289455
	**Normalized absorbances:**													
1	Baringo	0.076086	0.092413	0.111072	0.13015	0.146122	0.155197	0.154784	0.145099	0.129651	0.112884	0.097509	0.791868	1
2	Bomet	0.076086	0.092413	0.111072	0.13015	0.146122	0.155197	0.154784	0.145099	0.129651	0.112884	0.097509	0.791868	1
3	Elementaita	0.076765	0.093476	0.113251	0.13313	0.149112	0.157834	0.157042	0.147064	0.131194	0.113656	0.097691	0.764801	1
4	Emali	0.076765	0.093476	0.113251	0.13313	0.149112	0.157834	0.157042	0.147064	0.131194	0.113656	0.097691	0.764801	1
5	Gachika	0.070578	0.087205	0.106357	0.125739	0.14152	0.150253	0.149875	0.140456	0.125075	0.108021	0.092608	0.751337	1
6	Kihato	0.073396	0.090587	0.110846	0.130666	0.146257	0.154821	0.154485	0.14526	0.129495	0.111252	0.094867	0.784959	1
7	Likii	0.068492	0.084792	0.103238	0.121379	0.136044	0.144022	0.143572	0.135274	0.12166	0.106044	0.091472	0.762356	1
8	Loitoktok	0.083471	0.100932	0.120604	0.139834	0.155891	0.165731	0.166048	0.156426	0.140555	0.123312	0.107935	0.86648	1
9	Mahigaini	0.070795	0.087178	0.105828	0.124207	0.139347	0.147944	0.147281	0.137767	0.122679	0.105911	0.090479	0.745623	1
10	Maragua	0.069228	0.0855	0.104067	0.122658	0.137648	0.145743	0.144939	0.13552	0.120488	0.103925	0.089073	0.765126	1
11	Mbooni	0.072378	0.089896	0.109326	0.128277	0.143578	0.151511	0.150246	0.140869	0.126022	0.108799	0.092521	0.744938	1
12	Meru	0.073199	0.089622	0.109023	0.128269	0.143653	0.151868	0.150912	0.141184	0.125404	0.108063	0.092682	0.743765	1
13	Muhotetu	0.068779	0.085425	0.104345	0.123079	0.138299	0.146957	0.146549	0.13697	0.121585	0.104537	0.088912	0.733536	1
14	MUKURWE-INI-B	0.068582	0.084946	0.103913	0.122743	0.137551	0.145193	0.144281	0.135295	0.120779	0.104366	0.089407	0.755529	1
15	Mwea	0.086049	0.105747	0.126468	0.146851	0.164949	0.176035	0.17584	0.165097	0.148407	0.129839	0.112638	0.869369	1
16	Ruiri	0.06941	0.085769	0.104352	0.123208	0.138594	0.146772	0.146102	0.137067	0.122429	0.105869	0.09024	0.742919	1
17	Sipili	0.068858	0.08513	0.104118	0.123409	0.139454	0.148156	0.146976	0.136845	0.121072	0.103953	0.088655	0.743774	1
18	Sweet-Waters	0.071385	0.089421	0.109818	0.129353	0.14441	0.15242	0.151397	0.141481	0.126008	0.109027	0.093337	0.753881	1
19	Thika	0.072008	0.088784	0.108317	0.12755	0.142955	0.15163	0.151208	0.141611	0.126106	0.109012	0.093707	0.740654	1

ATR-FTIR Data from the end of the study

	**Mar., 2018 baselined absorbances**													
	**Wavenumbers:**	3019.824	3017.785	3015.746	3013.707	3011.668	3009.629	3007.59	3005.551	3003.512	3001.473	2999.434	2921.95	1743.383
1	Baringo	0.019856	0.024522	0.029961	0.035463	0.039965	0.04247	0.042373	0.039741	0.035351	0.030411	0.025993	0.218569	0.292513
2	Bomet	0.019699	0.024545	0.030199	0.035737	0.040219	0.042692	0.042336	0.03929	0.034715	0.029806	0.02539	0.21263	0.286928
3	Elementaita	0.019814	0.024579	0.029977	0.035535	0.040157	0.042693	0.042497	0.039658	0.03512	0.030114	0.025558	0.213503	0.288053
4	Emali	0.01982	0.024648	0.030256	0.035817	0.040353	0.042848	0.042607	0.039781	0.035347	0.03045	0.026025	0.215162	0.289738
5	Eq-Nanyuki	0.01912	0.023659	0.028797	0.034031	0.03852	0.041157	0.0411	0.038417	0.034075	0.029332	0.025061	0.219149	0.289031
6	Gachika	0.019707	0.024426	0.029784	0.03501	0.039194	0.0415	0.041278	0.03852	0.034131	0.029314	0.025094	0.217021	0.281386
7	Kab-Nyeri	0.020105	0.025034	0.030784	0.036457	0.040959	0.043395	0.043133	0.040295	0.035811	0.030819	0.026231	0.214025	0.289819
8	Kihato	0.020654	0.025296	0.030553	0.035873	0.040295	0.042757	0.042626	0.040014	0.035675	0.030772	0.026396	0.219401	0.289148
9	Likii	0.019357	0.024153	0.029641	0.03503	0.039271	0.041501	0.041213	0.03855	0.034341	0.029611	0.025307	0.2203	0.29209
10	Loitoktok	0.020074	0.024913	0.030296	0.035683	0.04013	0.042534	0.042311	0.039603	0.035247	0.030339	0.025792	0.212447	0.2877
11	Mahigaini	0.020417	0.025267	0.030711	0.036143	0.040564	0.042863	0.042505	0.039687	0.035143	0.03008	0.025563	0.214701	0.287699
12	Makuyu	0.01908	0.023807	0.029209	0.034474	0.038552	0.040725	0.040525	0.037948	0.033831	0.029252	0.02501	0.218051	0.288967
13	Maragua	0.019425	0.024237	0.029564	0.034837	0.039344	0.041986	0.041892	0.039216	0.034945	0.03017	0.025835	0.217177	0.290293
14	Matanya	0.019828	0.024668	0.03015	0.035598	0.040016	0.042405	0.042128	0.039372	0.035051	0.030165	0.025624	0.214347	0.290056
15	Matuu	0.020433	0.025019	0.030256	0.035656	0.040184	0.042745	0.042614	0.039848	0.035392	0.030479	0.026033	0.217799	0.282254
16	Mbooni	0.02003	0.024634	0.029867	0.035223	0.039689	0.042217	0.042105	0.039374	0.034962	0.03014	0.025823	0.2153	0.285193
17	Meru	0.019889	0.024684	0.030195	0.035706	0.040216	0.042646	0.04228	0.039408	0.035016	0.030163	0.025728	0.215543	0.28955
18	Muhotetu	0.019207	0.023777	0.028888	0.03419	0.038688	0.041217	0.041071	0.038426	0.034168	0.029395	0.02502	0.219549	0.289623
19	MUKURWE-INI-A	0.019924	0.024722	0.030182	0.035589	0.040117	0.042748	0.042573	0.039668	0.035153	0.030234	0.025762	0.215014	0.292441
20	MUKURWE-INI-B	0.019763	0.024565	0.030156	0.035797	0.040298	0.042653	0.042388	0.039628	0.035154	0.030085	0.025468	0.212769	0.290658
21	Mutalani	0.019964	0.024643	0.030005	0.035432	0.039825	0.042187	0.041974	0.039246	0.034822	0.02987	0.025367	0.215947	0.287935
22	Mwea	0.019316	0.023875	0.029066	0.034283	0.038516	0.040825	0.040798	0.038416	0.034229	0.029442	0.025107	0.22173	0.288945
23	Nakuru	0.020046	0.024808	0.030228	0.03566	0.040099	0.042566	0.042338	0.039454	0.034946	0.030067	0.025714	0.217037	0.291378
24	Naro-Moru	0.020223	0.025105	0.030673	0.036237	0.040781	0.043226	0.042956	0.040084	0.035526	0.030542	0.02599	0.212297	0.281239
25	Nyeri	0.0195	0.024424	0.030051	0.035573	0.040017	0.042412	0.042192	0.039463	0.035012	0.02992	0.02529	0.21291	0.289211
26	Ruiri	0.019909	0.024716	0.030199	0.035772	0.040412	0.043031	0.04297	0.040213	0.035478	0.030147	0.025447	0.213225	0.291302
27	Sagana	0.019788	0.024762	0.03042	0.036025	0.040582	0.042978	0.04265	0.039816	0.035314	0.030273	0.025745	0.213513	0.289308
28	Sipili	0.020039	0.024678	0.029765	0.035092	0.03972	0.042323	0.042232	0.039603	0.035254	0.030271	0.025612	0.215097	0.289853
29	Sweet-Waters	0.019419	0.024206	0.029711	0.035029	0.039305	0.041666	0.041494	0.038956	0.034808	0.029984	0.025514	0.216881	0.288557
30	Thika	0.023247	0.028062	0.033556	0.038897	0.043216	0.045602	0.045327	0.042548	0.038201	0.033392	0.029029	0.221377	0.243766
30	Thika (rep)	0.023789	0.028321	0.033432	0.038734	0.043239	0.045828	0.04582	0.043229	0.038907	0.033993	0.029397	0.220235	0.242704
		Thika seeds had all grown moldy; data not used												
	**Normalized absorbances:**													
1	Baringo	0.067881	0.083832	0.102426	0.121236	0.136626	0.14519	0.144859	0.135861	0.120853	0.103965	0.088861	0.747211	1
2	Bomet	0.068655	0.085544	0.105249	0.12455	0.140171	0.14879	0.147549	0.136933	0.120989	0.10388	0.088489	0.741057	1
3	Elementaita	0.068786	0.085328	0.104068	0.123363	0.139408	0.148212	0.147532	0.137676	0.121922	0.104543	0.088727	0.741193	1
4	Emali	0.068407	0.08507	0.104425	0.123619	0.139274	0.147885	0.147054	0.1373	0.121996	0.105095	0.089823	0.742609	1
5	Eq-Nanyuki	0.066152	0.081856	0.099633	0.117742	0.133273	0.142396	0.142199	0.132917	0.117894	0.101484	0.086707	0.75822	1
6	Gachika	0.070035	0.086806	0.105847	0.12442	0.139289	0.147484	0.146695	0.136894	0.121296	0.104177	0.08918	0.771257	1
7	Kab-Nyeri	0.069371	0.086378	0.106218	0.125792	0.141326	0.149731	0.148827	0.139035	0.123563	0.106339	0.090508	0.738478	1
8	Kihato	0.071431	0.087485	0.105666	0.124064	0.139358	0.147872	0.147419	0.138386	0.12338	0.106423	0.091289	0.758784	1
9	Likii	0.066271	0.08269	0.101479	0.119929	0.134448	0.142083	0.141097	0.13198	0.11757	0.101376	0.086641	0.75422	1
10	Loitoktok	0.069774	0.086594	0.105304	0.124029	0.139486	0.147842	0.147066	0.137654	0.122513	0.105454	0.089649	0.738432	1
11	Mahigaini	0.070967	0.087824	0.106747	0.125628	0.140995	0.148986	0.147741	0.137946	0.122152	0.104554	0.088853	0.74627	1
12	Makuyu	0.066028	0.082387	0.101081	0.119301	0.133413	0.140933	0.140241	0.131323	0.117076	0.10123	0.08655	0.754588	1
13	Maragua	0.066915	0.083492	0.101842	0.120006	0.135532	0.144633	0.144309	0.135091	0.120378	0.103929	0.088996	0.74813	1
14	Matanya	0.068359	0.085046	0.103945	0.122728	0.13796	0.146196	0.145241	0.135739	0.120842	0.103997	0.088342	0.738985	1
15	Matuu	0.072392	0.08864	0.107194	0.126326	0.142368	0.151442	0.150977	0.141178	0.125391	0.107984	0.092233	0.771642	1
16	Mbooni	0.070233	0.086377	0.104726	0.123506	0.139165	0.14803	0.147637	0.138061	0.122591	0.105683	0.090546	0.754927	1
17	Meru	0.068689	0.08525	0.104283	0.123315	0.138891	0.147284	0.14602	0.136101	0.120932	0.104172	0.088855	0.744407	1
18	Muhotetu	0.066317	0.082096	0.099743	0.11805	0.133581	0.142313	0.141808	0.132676	0.117974	0.101494	0.086388	0.758051	1
19	MUKURWE-INI-A	0.06813	0.084537	0.103207	0.121696	0.13718	0.146176	0.145578	0.135644	0.120205	0.103385	0.088093	0.735239	1
20	MUKURWE-INI-B	0.067994	0.084515	0.103751	0.123158	0.138644	0.146746	0.145835	0.136339	0.120946	0.103507	0.087622	0.732025	1
21	Mutalani	0.069335	0.085585	0.104208	0.123056	0.138312	0.146516	0.145776	0.136302	0.120937	0.103739	0.0881	0.749985	1
22	Mwea	0.06685	0.082628	0.100594	0.118649	0.133299	0.14129	0.141196	0.132953	0.118462	0.101895	0.086892	0.767378	1
23	Nakuru	0.068797	0.08514	0.103742	0.122384	0.137618	0.146085	0.145303	0.135405	0.119934	0.103189	0.08825	0.744864	1
24	Naro-Moru	0.071907	0.089266	0.109064	0.128848	0.145005	0.153698	0.152738	0.142526	0.12632	0.108598	0.092413	0.754863	1
25	Nyeri	0.067425	0.08445	0.103907	0.123	0.138366	0.146647	0.145887	0.136451	0.12106	0.103454	0.087445	0.736175	1
26	Ruiri	0.068345	0.084847	0.103669	0.1228	0.138729	0.14772	0.14751	0.138046	0.121791	0.103491	0.087356	0.731972	1
27	Sagana	0.068398	0.08559	0.105147	0.124521	0.140273	0.148554	0.147421	0.137625	0.122064	0.104639	0.088988	0.738013	1
28	Sipili	0.069135	0.08514	0.10269	0.121068	0.137035	0.146015	0.145701	0.136631	0.121627	0.104436	0.088362	0.74209	1
29	Sweet-Waters	0.067297	0.083886	0.102964	0.121394	0.136212	0.144394	0.143798	0.135003	0.120628	0.10391	0.088419	0.751605	1

Table 4a – Example GC-MS data with retention times, area counts, and compound identity.

GC-MS Data													
Example workup of the GC-MS data for Baringo from solvent-extracted oil for March, 2018													
Peak #	Ret Time	Area	% Area			Baringo-re_3_18							
1	22.164	288558	0.034	C14:0		Ion 292.30 (292.00 to 293.00): Baringo-re_3_18.D\data.ms							
2	28.081	105163	0.013	C16:2		Peak #	Ret Time	Type	Width	Area	Start Time	End Time	
3	28.176	16061	0.002	C16:1		1	34.178	M	0.068	262694	34.093	34.258	0.32983
4	28.802	36039013	4.302	C16:0		Ion 296.30 (296.00 to 297.00): Baringo-re_3_18.D\data.ms							
5	31.083	52605	0.006	C17:2		1	34.181	M	0.062	533759	34.111	34.398	
6	31.152	32748	0.004	C17:1						796453			
7	31.934	181001	0.022	C17:0									
8	34.051	6.18E+08	73.780	C18:2									
9	34.176	1.21E+08	14.420	C18:1/18:3									
10	34.323	11902525	1.421	C18:1									
11	34.962	40297198	4.810	C18:0									
12	36.71	1139067	0.136	C18:2									
13	39.732	496604	0.059	C20:2									
14	39.886	6366325	0.760	C20:1									
15	40.645	1799203	0.215	C20:0									
16	43.474	23674	0.003	C21:2?									
17	43.5	11994	0.001	C21:1?									
18	43.853	11679	0.001	C21:0?									
19	45.892	50910	0.006	C22:2?									
20	46.12	24209	0.003	C22:1?									
21	46.496	16678	0.002	C22:0?									
		8.38E+08											

Table 4b – Summary of GC-MS relative percentage of each component at each location.

Raw GC-MS FAME percentages for March by solvent extraction																
Site #	Site	C14:0	C16:2	C16:1	C16:0	C17:2	C17:1	C17:0	C18:2a	C18:3	C18:1a	C18:1b	C18:0	C18:2b	C20:2	C20:1	C20:0
1	Baringo	0.034	0.013	0.002	4.302	0.006	0.004	0.022	73.780	4.756	9.664	1.421	4.810	0.136	0.059	0.760	0.215
2	Bomet	0.048	0.007	0.004	3.444	0.011	0.004	0.031	76.381	7.139	5.741	0.803	4.475	0.118	0.257	1.030	0.398
3	Element	0.014	0.001	0.000	3.183	0.000	0.000	0.006	78.346	5.015	7.408	0.995	4.235	0.063	0.060	0.524	0.134
4	Emali	0.018	0.003	0.002	3.231	0.002	0.001	0.009	79.019	3.870	7.932	0.855	4.026	0.070	0.089	0.627	0.181
5	Eq-Nanyuki	0.017	0.002	0.000	3.420	0.001	0.000	0.007	74.200	3.279	11.101	0.984	6.120	0.058	0.034	0.579	0.183
6	Gachika	0.060	0.018	0.017	5.024	0.002	0.001	0.008	76.960	5.699	5.023	1.234	4.653	0.063	0.171	0.597	0.414
7	Kab-Nyeri	0.020	0.001	0.000	3.113	0.001	0.000	0.010	78.976	5.584	6.716	0.900	3.889	0.046	0.063	0.487	0.156
8	Kihato	0.022	0.003	0.002	2.265	0.006	0.004	0.004	77.130	5.883	10.247	1.184	3.089	0.008	0.016	0.029	0.024
9	Likii	0.012	0.001	0.000	5.527	0.000	0.001	0.007	72.211	7.296	9.090	1.218	4.232	0.041	0.020	0.257	0.068
10	Loitoktok	0.033	0.006	0.003	4.246	0.006	0.001	0.017	76.503	6.168	6.720	1.182	3.741	0.100	0.150	0.873	0.241
11	Mahigaini	0.016	0.001	0.000	3.017	0.001	0.000	0.004	77.721	9.574	4.995	1.133	3.326	0.021	0.013	0.103	0.045
12	Makuyu	0.015	0.003	0.002	3.516	0.001	0.002	0.002	71.635	9.116	10.406	1.754	3.289	0.016	0.028	0.120	0.046
13	Maragua	0.029	0.001	0.001	3.739	0.001	0.000	0.009	73.566	6.585	9.588	0.976	4.550	0.060	0.052	0.628	0.186
14	Matanya	0.006	0.001	0.000	2.625	0.001	0.000	0.002	78.833	6.212	7.508	1.584	3.029	0.018	0.008	0.105	0.028
15	Matuu	0.052	0.012	0.005	3.635	0.005	0.002	0.017	75.385	4.406	9.004	1.019	5.260	0.077	0.082	0.721	0.260
16	Mbooni	0.010	0.000	0.000	2.811	0.000	0.000	0.002	79.083	4.905	7.494	1.143	3.790	0.038	0.050	0.482	0.166
17	Meru	0.036	0.003	0.002	3.665	0.005	0.001	0.014	80.935	3.021	5.478	0.891	4.437	0.085	0.187	0.880	0.322
18	Muhotetu	0.029	0.002	0.000	4.093	0.001	0.000	0.009	75.283	3.066	10.021	1.266	5.340	0.064	0.055	0.550	0.206
19	MUKUR-A	0.023	0.003	0.000	3.319	0.001	0.000	0.009	75.396	6.135	8.390	1.006	5.392	0.056	0.017	0.177	0.061
20	MUKUR-B	0.013	0.001	0.000	3.461	0.001	0.000	0.004	78.365	5.912	7.499	1.738	2.715	0.053	0.019	0.178	0.032
21	Mutalani	0.028	0.008	0.005	4.311	0.002	0.001	0.010	77.721	4.538	6.189	1.098	4.597	0.059	0.153	0.820	0.439
22	Mwea	0.006	0.001	0.000	3.081	0.000	0.000	0.004	72.637	4.375	12.607	1.607	5.398	0.036	0.008	0.160	0.057
23	Nakuru	0.059	0.014	0.006	4.288	0.008	0.003	0.017	73.509	6.485	8.379	1.240	5.093	0.083	0.070	0.489	0.177
24	Naro-Moru	0.010	0.001	0.000	2.232	0.000	0.000	0.002	78.791	6.050	7.411	1.699	3.579	0.039	0.014	0.128	0.033
25	Nyeri	0.012	0.000	0.000	2.654	0.000	0.000	0.003	77.621	7.335	6.537	1.299	3.972	0.040	0.037	0.362	0.109
26	Ruiri	0.056	0.006	0.004	2.995	0.008	0.002	0.016	79.782	4.537	5.836	0.824	4.352	0.234	0.173	0.823	0.295
27	Sagana	0.009	0.001	0.000	2.862	0.000	0.000	0.005	80.363	5.071	6.014	1.642	3.573	0.049	0.031	0.295	0.065
28	Sipili	0.048	0.018	0.006	4.600	0.015	0.008	0.036	76.799	4.688	6.830	0.883	4.356	0.112	0.198	0.971	0.344
29	Sweetwater	0.006	0.000	0.000	2.798	0.000	0.000	0.003	73.729	5.790	11.050	1.395	4.850	0.046	0.014	0.235	0.069
30	Thika	0.020	0.003	0.001	3.637	0.002	0.001	0.012	72.981	9.742	5.819	1.199	5.652	0.071	0.046	0.561	0.238

Table 5 – GIS interpolated data and the corresponding z-scores for the initial normalized absorbance, precipitation and temperature at each site.

		GIS Interpolated		Initial	Final	z-scores of data		
	Location	Avg P/yr	Avg T/yr	Normed A	Normed A	Initial-A	Avg P/yr	Avg T/yr
1	Baringo-Kabarnet	112	16.9	0.1537	0.1452	1.28835	1.41266	-0.76586
2	Bomet	103	17.4	0.1529	0.1488	1.09515	0.99898	-0.41435
3	Elementaita	57	17.5	0.1484	0.1482	0.04531	-1.11542	-0.3583
4	Emali	58	21.6	0.1457	0.1479	-0.59068	-1.06946	2.13281
5	Equator-Nanyuki	67	16.1	0.1470	0.1424	-0.28947	-0.65577	-1.2447
6	Gachika	83	17.8	0.1503	0.1475	0.49082	0.07967	-0.17998
7	Kabiruini-Nyeri	77	17.6	0.1498	0.1497	0.37283	-0.19612	-0.33794
8	Kihato	65	16.5	0.1486	0.1479	0.09301	-0.7477	-0.98489
9	Likii	67	16.3	0.1551	0.1421	1.6161	-0.65577	-1.11737
10	Loitoktok	85	17.9	0.1414	0.1478	-1.5968	0.1716	-0.12905
11	Mahigaini	95	19.9	0.1511	0.1490	0.68086	0.63126	1.09865
12	Makuyu	83	19.9	0.1420	0.1409	-1.47129	0.07967	1.10886
13	Maragua	93	20.0	0.1494	0.1446	0.27556	0.53932	1.13435
14	Matanya	64	16.6	0.1438	0.1462	-1.03337	-0.79367	-0.90848
15	Matuu	60	21.0	0.1467	0.1514	-0.35044	-0.97753	1.75074
16	Mbooni	96	17.7	0.1434	0.1480	-1.14577	0.67722	-0.24624
17	Meru	151	18.4	0.1521	0.1473	0.92063	3.20531	0.14604
18	Muhotetu	60	15.7	0.1535	0.1423	1.24449	-0.97753	-1.46887
19	MUKURWE-INI-A	110	17.6	0.1481	0.1462	-0.04274	1.32073	-0.28188
20	MUKURWE-INI-B	110	17.6	0.1447	0.1467	-0.83218	1.32073	-0.28188
21	Mutulani	54	19.3	0.1483	0.1465	0.00653	-1.25332	0.71658
22	Mwea	101	19.9	0.1460	0.1413	-0.53318	0.90705	1.07322
23	Nakuru	76	17.7	0.1531	0.1461	1.13712	-0.24208	-0.24624
24	Naro_Moru	69	16.3	0.1400	0.1537	-1.93327	-0.56384	-1.12751
25	Nyeri	81	17.7	0.1458	0.1466	-0.57014	-0.01226	-0.25132
26	Ruiru	66	19.4	0.1522	0.1477	0.9376	-0.70174	0.81341
27	Sagana	91	20.8	0.1414	0.1486	-1.60663	0.44739	1.64376
28	Sipili	67	16.3	0.1504	0.1460	0.51403	-0.65577	-1.13772
29	Sweet_Waters	64	16.4	0.1548	0.1444	1.55447	-0.79367	-1.04095
30	Thika	73	19.6	0.1471	(moldy)	-0.27694	-0.37998	0.90511

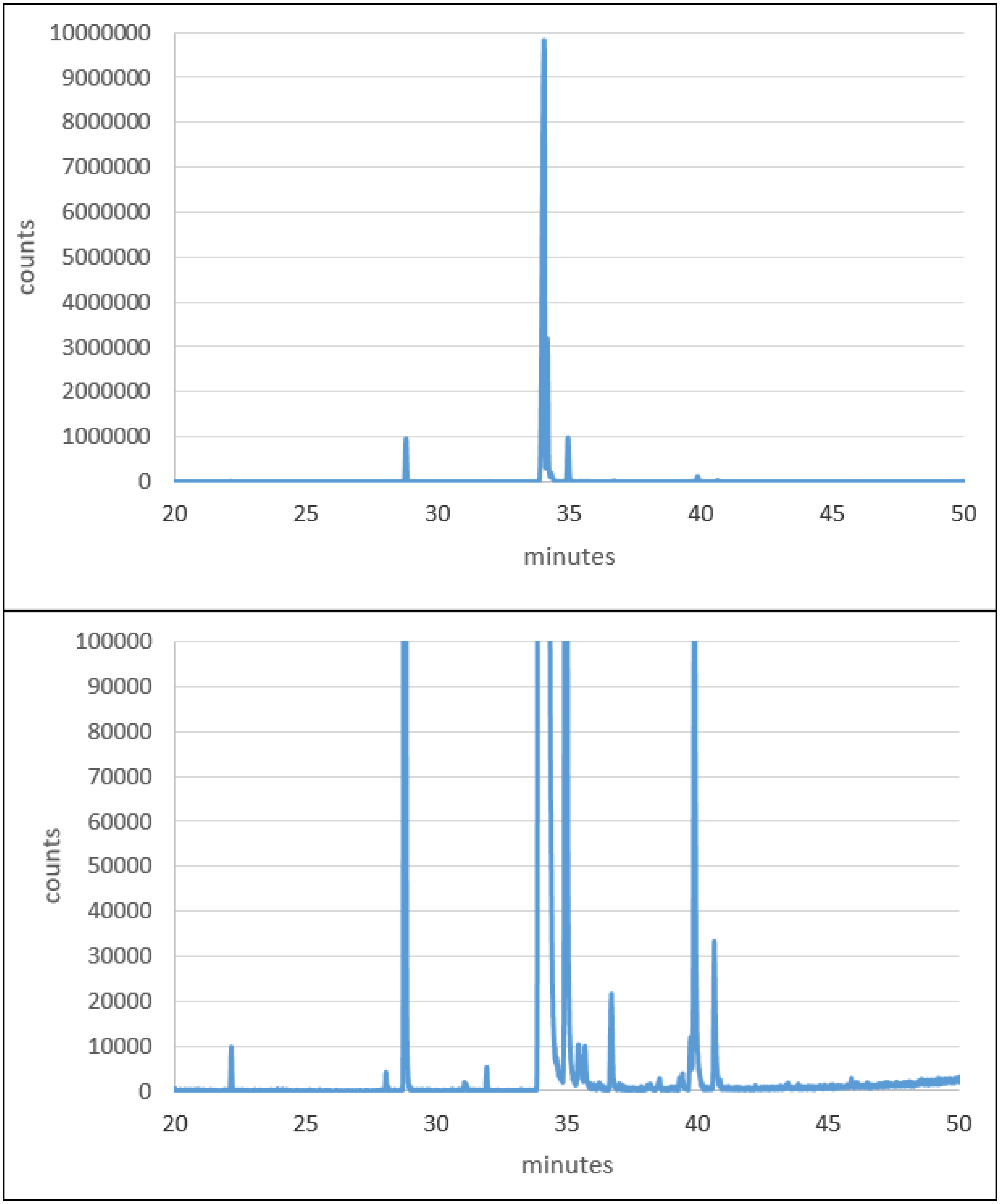

Fig. 1 – Example TICs using oil from a Baringo nut, sampled and analyzed during December 2017. Lower plot is magnified by 100X to reveal the FAME peaks with low counts.

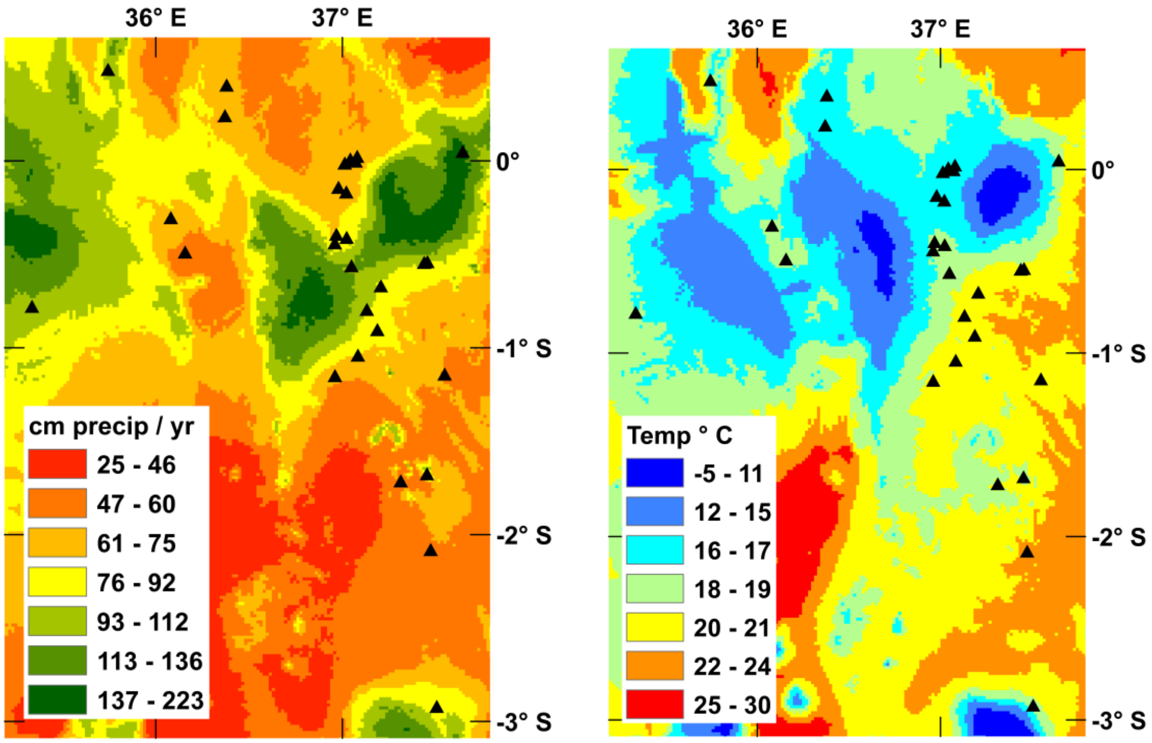

Fig. 2a – Interpolated map of mean annual precipitation (triangles are the study sites); and Fig. 2b – Interpolated map of mean annual temperature, developed using ArcGIS v 10.4.1 with Spatial Analyst Extension and 1 km^2 resolution climate data from http://www.worldclim.org/

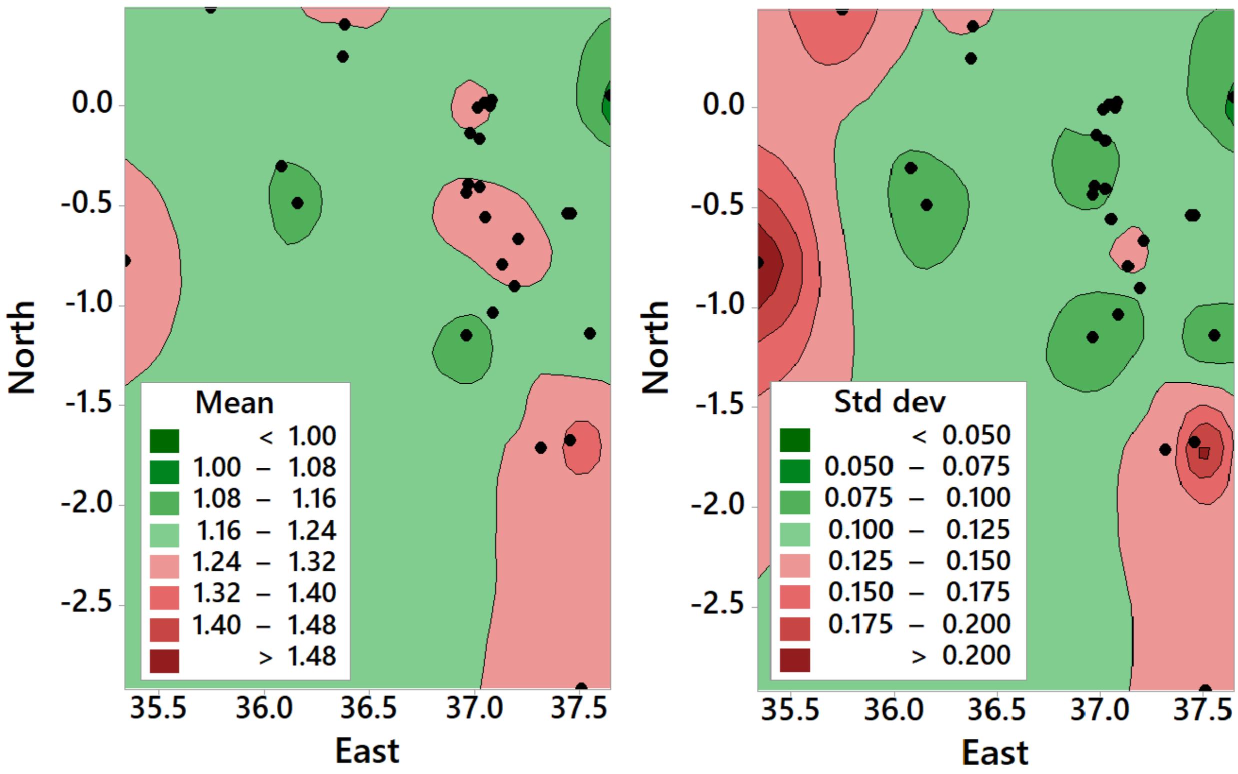

Fig. 3a – Interpolated maps of the morphological variation (length to width ratio) and (3b) its standard deviation, illustrating a relationship (especially in the variation of the ratio) with the geography and/or local climate. (Maps created using Minitab 18 and data measured from photographs using ImageJ.)

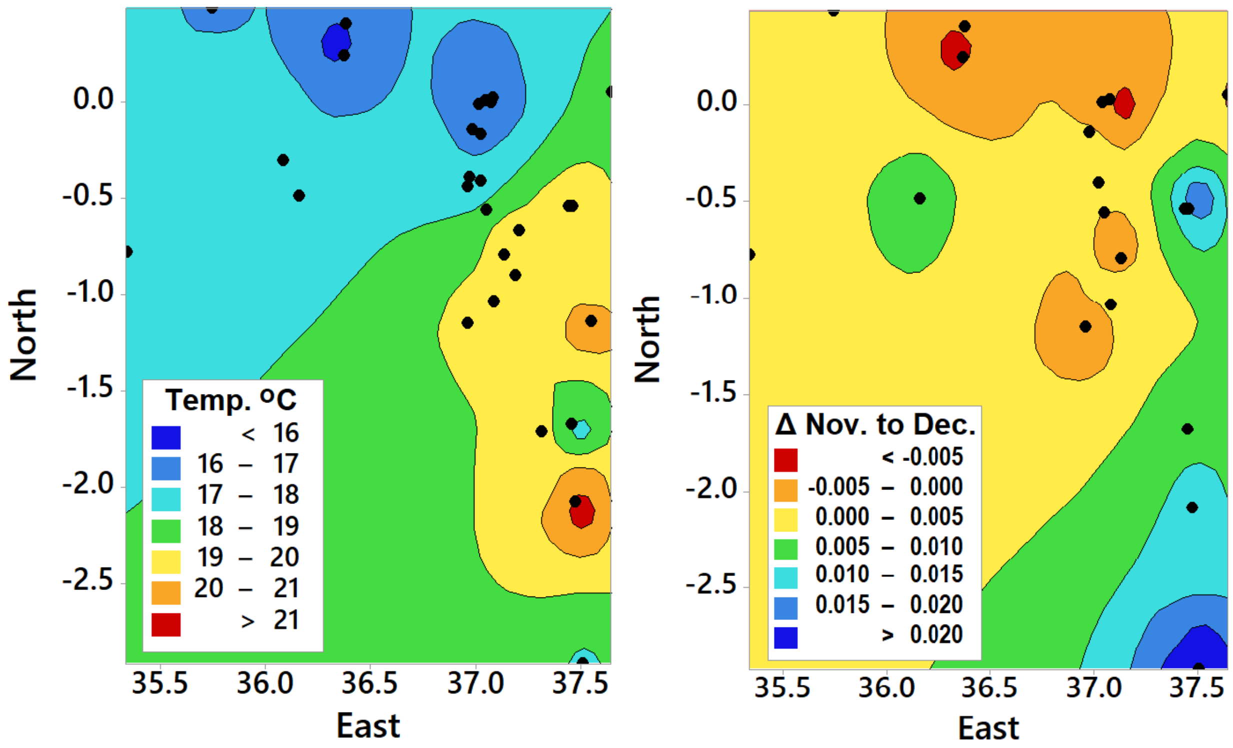

Fig. 4a – Interpolated maps of the mean annual temperature at each site and (4b) the gain (loss) in the poly-unsaturation proxy at 3009.6 cm^{-1}, illustrating the greatest gains in unsaturation occurred with nuts placed in 0°C storage from sites that have the highest annual temperatures. (Maps created using Minitab 18 and temperature data from ArcGIS v 10.4.1 interpolations shown in Supplementary Material Figure 2b and Supplementary Table 5.)
